# Circulation of Toscana Virus in a Sample Population of Corsica, France

**DOI:** 10.3390/v11090817

**Published:** 2019-09-04

**Authors:** Shirley Masse, Nazli Ayhan, Lisandru Capai, Frédéric Bosseur, Xavier de Lamballerie, Rémi Charrel, Alessandra Falchi

**Affiliations:** 1 Université de Corse Pascal Paoli, EA7310 BIOSCOPE, 20250 Corte, France; 2 Unité des Virus Emergents (UVE: Aix Marseille Univ, IRD 190, INSERM 1207, IHU Méditerranée Infection), 13385 Marseille, France; 3 Sciences Pour l’Environnement—UMR CNRS 6134, Université de Corse, 20250 Corte, France

**Keywords:** Sandfly Fever Sicilian virus, seroprevalence, microneutralization, meningitis, arbovirus, arthropod-borne virus, *Phlebovirus*, *Phenuiviridae*, *Bunyavirales*, sandfly

## Abstract

Sandfly-borne phleboviruses pathogenic to humans, such as Toscana virus (TOSV) and Sandfly Fever Sicilian virus (SFSV), are endemic in the Mediterranean region. In France, several autochthonous cases of TOSV infection have been described, causing either meningitis or encephalitis. The aim of the present study was to investigate the seroprevalence of TOSV and SFSV antibodies in a healthy population from Corsica. In this cross-sectional study, participants were enrolled (i) from a medical staff at the University of Corsica and (ii) from general practitioners of the Corsican Sentinelles Network. The seroprevalence study was based on a virus microneutralization assay. A total of 240 sera were tested. Altogether, 54 sera (22.5%) were confirmed positive for TOSV antibodies, whereas none were positive for SFSV (0/240). The residential district of participants was significantly associated with TOSV seropositivity (*p* value = 0.005). The rate of the seropositivity against TOSV in our study suggests that the Corsican population is well exposed to the TOSV. These results encourage the implementation of a systematic surveillance system including entomological, microbiological, and medical aspects for the collection of better information on the diseases that are associated with phleboviruses in Corsica and beyond in the regions where these viruses are present.

## 1. Introduction

Sandfly-borne phleboviruses pathogenic to humans such as Toscana virus (TOSV) and Sandfly Fever Sicilian virus (SFSV) are endemic in the Mediterranean region [1,2]. Although most human infections are either asymptomatic or influenza-like syndromes, TOSV has emerged as outbreaks and sporadic cases of acute meningitis or meningoencephalitis. TOSV human cases have been reported in southern European countries such as Italy, Spain, Greece, Portugal, and France, including most of the Mediterranean islands [3]. SFSV frequently causes epidemics of febrile illness during the warm seasons [1] and is not neurotropic [4].

In France, the first case of TOSV infection was reported in a German tourist returning from the region of Marseille in southeastern France [5]. Since then, several autochthonous cases of TOSV infection in France have been described, causing either meningitis [6] or encephalitis [7]. Furthermore, myositis was reported as an additional clinical complication of TOSV infection [8]. Concerning SFSV, low seroprevalence rates of SFSV antibodies were recorded in southwestern France (2%) and in Marseille (1%) among blood donors [9].

In southeastern France, two seroprevalence studies conducted with blood donors reported TOSV rates ranging from 6.5% to 19% [3]. In Corsica, the presence of TOSV RNA was reported in *Phlebotomus perniciosus* sandflies, one of the major vectors of TOSV [10]. Genetic analysis showed that Corsican TOSV belongs to lineage A [9]. The seroprevalence of TOSV was introduced at 8.7% in 2007 among Corsican blood donors by using the ELISA method [11], although this technique lacks specificity and is prone to cross-reactions with other phleboviruses, and thus may overestimate human exposure compared with seroprevalence studies based on a neutralization assay. Moreover, a microneutralization (MN)-based seroprevalence assay, conducted in 2013–2014 in dog sera, showed a TOSV seropositivity of 3.9% in dogs on the eastern coast of Corsica [12].

To date. in Corsica the data on the circulation of TOSV are scarce and fragmentary, and the data on SFSV circulation in Corsica are absent. Despite TOSV being one of the most prevalent causes of viral central nervous system infection in regions where the sandfly vectors are present, it remains neglected because of the lack of awareness of both physicians and the general population, unlike other viruses such as Zika, Chikungunya, Dengue, or West Nile that have been popularized by the media. TOSV infections frequently remain undiagnosed because of their nonspecific symptoms and the underestimation of this virus’s large geographical spread in the Mediterranean basin. As a direct consequence, the diagnostic of TOSV is rarely prescribed when facing a patient presenting clinical manifestations. Our study aimed to measure the seroprevalence of TOSV and SFSV in a healthy population of Corsica concerning socio-demographic and lifestyle.

## 2. Materials and Methods

### 2.1. Study Area

The study was conducted in the French Mediterranean island of Corsica. It consists of two administrative departments (Haute-Corse and Corse-du-Sud) and five districts (Ajaccio, Bastia, Calvi, Corte, Sartène) including 365 counties.

### 2.2. The Study

In this cross-sectional study, participants were enrolled from medical staff at the University of Corsica Pasquale Paoli (UCPP) from January 2017 to January 2019 and from general practitioners (GPs) of the “Sentinelles” network from June 2017 to September 2017. The UCPP, the only university present in the island, is a multidisciplinary institution including eight faculties, institutes, and schools (https://www.universita.corsica/en/). The Corsican GP Sentinelles network, a part of the French Sentinelles Network [13] (http://www.sentiweb.fr), is a real-time epidemiologic surveillance system based on volunteer GPs located throughout Corsica.

Participants were included in the study if they declared living in Corsica for at least six months in the year of the study enrollment. A blood sample and a questionnaire were obtained from each participant. The blood samples were collected by using a Safety-Lancet on a cleansed finger puncture. The questionnaire recorded information about socio-demographical variables (age, sex, residential district, education, occupation, travels, and type of dwelling), clinical factors (presence of chronic diseases, organ transplantation, blood transfusion), and contact with animals (hunting and breeding).

### 2.3. Serological Analyses

The virus MN assay of this study was adapted from the protocol described previously [14]. Sera were tested in parallel for TOSV (strain MRS2010–4319501) and SFSV (strain Sabin). The MN assay was performed in 96-well microtiter plates using Vero cells. Briefly, two-fold serial dilutions of 50 μL serum aliquots were mixed with an equal volume of 100 TCID50 (Tissue Culture Infective Dose producing pathological change in 50% of the cell culture inoculated) of viruses into 96-well plates, providing two-fold final dilutions between 1:20 and 1:160. Controls consisted of each serum (1:10) with Vero cells but without the virus. After five days (for TOSV) and six days (for SFSV), the microplates were read and the presence (neutralization titer at 20, 40, 80, and 160) or absence (no neutralization) of the cytopathic effect was noted. The cutoff value for positivity was set at titre ≥40 as previously described using the same technique [15,16].

### 2.4. Sample Size

The sample size was calculated according to previously described methods [17]. A sample size of 126 was calculated assuming an a priori 9% anti-TOSV and anti-SFSV specific IgG seroprevalence [9,11,18], a confidence in the estimate of 95%, a maximum allowable error in the prevalence of 5%, and a Corsican population size of 330,455 habitants (based on the latest French census data).

### 2.5. Statistical Analyses

Descriptive statistic methods were performed for all variables. Continuous data were reported as medians. All categorical data were reported as percentages. 

All variables were tested in a univariate analysis. Odds ratios (ORs) with 95% confidence intervals (CIs) were obtained and *p* values < 0.05 were considered to be significant. Then, variables with a *p* value below 0.2 were tested in multivariate analyses, with a random effect at the population level. Goodness of fit was calculated for each model, and the model with the lowest Akaike Information Criterion was considered to have the best fit. Age was included in the models as a categorized variable as 0–20 years, 20–30 years, 30–40 years, 40–60 years, and 60–99 years. All statistical analyses were performed using the R program (http://www.r-project.org) [19].

### 2.6. Ethics

The study was approved by the ad hoc ethics committee (Human Research Ethics Committee Sud Méditérannée II (11 January 2017)) and the project identification code is #2016-A01000-51 

All the collected data and the questionnaire were checked and validated by the data protection officer of the University of Corsica. All participants included were voluntary and non-remunerated. They were informed that samples would be used for seroprevalence studies by a letter of information, following which all participants signed a consent form.

## 3. Results

**Figure 1:**
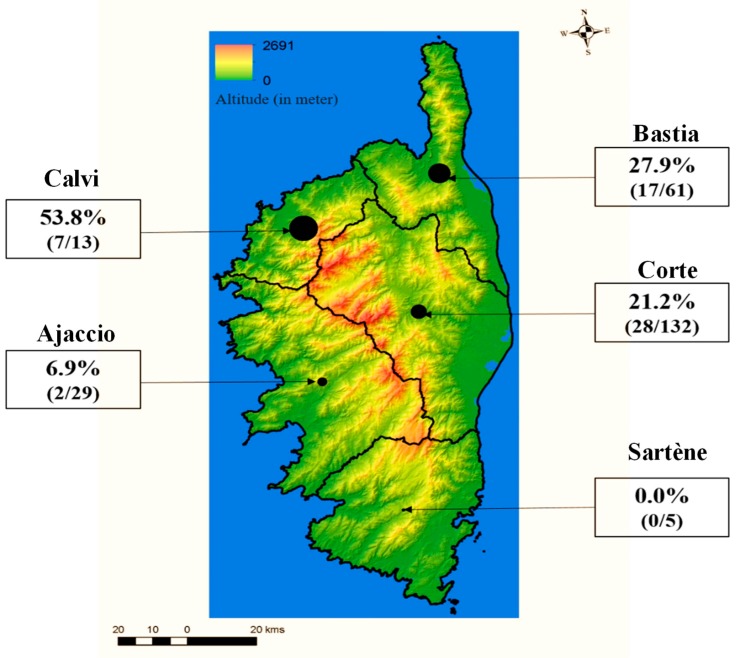
Distribution of Toscana virus (TOSV) seroprevalence among districts in Corsica.

The study sample of 240 participants consisted of 142 women (59%) and 98 men (41%). Age ranged from 9 to 84 years with a median age of 24.5 years. Among the 240 participants, 25 (10%) were enrolled by GPs and 215 (90%) by the medical staff of UCPP. All participant sera were tested for the presence of neutralizing antibodies against TOSV and SFSV. They consisted of 29 (12.0%), 61 (25.5%), 13 (5.5%), 132 (55.0%), and 5 (2.0%) sera collected from the districts of Ajaccio, Bastia, Calvi, Corte, and Sartene, respectively (Figure 1). The characteristics and seroprevalence of the study population are presented in Table 1 and Figure 1.

Altogether, 54 sera were confirmed for containing antibodies against TOSV (22.5%) and none were confirmed against SFSV (Table 1). The TOSV-positive sera belonged to participants aged from 17 to 78 years with a median of 24 years, with a predominance of women (60%; 32 out of 54) (Table 1).

There was no significant difference in the distribution of TOSV according to sex (*p* = 0.987). Although it was not statistically significant, the seroprevalence increased with age from 19.6% in the 0–20 years age group to 37.5% in the 60–99 years age group (*p* = 0.500). Eleven of 26 sera (19.6%) of the 0–20 years age group, confirmed for TOSV, belonged to one 17 year old participant, two 18 year old participants, and eight 19 year old participants. Six of the 16 sera (37.5%) confirmed for TOSV of the 60–99 years age group belonged to two participants aged 65 and 67 years, and four aged 73, 75, 76, and 78 years.

The residential district of participants was significantly associated with TOSV seropositivity, ranging from 0% in Sartene (south) to 53.8% in Calvi (northwest coast) (*p* = 0.005) (Table 1 and Figure 1). This association was confirmed by the multivariate analysis (*p* = 0.005) (Table 2). No other risk factors were identified for TOSV seropositivity.

## 4. Discussion

Serological surveys have reported that TOSV is endemic in the Mediterranean Basin [20], ranging in the general population from 5% in Spain [21] to 51.7% in Cyprus [22]. The TOSV seroprevalence observed in our study was close to 26.7% and 33% observed in central Italy [23,24] and Sicily [18], respectively.

This study reports new data on rates of TOSV- and SFSV-neutralizing antibodies in human sera collected in a sample of healthy individuals on the island of Corsica. The absence of seropositivity to SFSV seems to confirm the little to no circulation of this virus in Corsica [9].

In agreement with previous studies [18,25,26,27], we observed that TOSV seroprevalence increased with age, demonstrating that the Corsican population is exposed to TOSV throughout life. This is also important clinical information, as adults have a higher risk of neurological complications due to TOSV than children [25]. The overall TOSV antibodies rate observed in our study suggests a more intense circulation of TOSV in Corsica, with a rate significantly higher than the 8.7% reported in Corsica in 2007 from blood donors [11]. A study performed in 2011 on 2625 sera collected from blood donors in France showed ELISA and MN rates of 7.3% and 2.11%, respectively, in southeastern France. For the subset corresponding to Corsica (*n* = 538), the observed values were not higher than those in continental southeastern regions (data not shown) [28] Moreover, the present study could contribute to the identification of areas where the TOSV circulates.

We observed that the circulation of TOSV was not homogeneous among the five residence districts, with a high seroprevalence reported in the northwest coast (Calvi district). Data on the species of sandflies of the genus *Phlebotomus* in the island are rare. Historical records characterize *P. mascitii*, *P. perniciosus,* and *Sergentomyia minuta* as only existing in Corsica at altitudes lower than 800 m [29]. *Phlebotomus* (*Transphlebotomus*) *mascittii* were observed during winter in a railway tunnel of the island [30]. Canine leishmaniasis is vectored almost exclusively by *P. perniciosus* in Corsica, the same vector that also transmits TOSV. A recent canine leishmaniasis risk map in southern France indicated that *P. perniciosus* could be quite widespread in the island [31]. Interestingly, a recent study conducted in Corsica established that the number of cases of canine leishmaniasis, one of the most important in southern France with 21 to 50 cases per year, has been stable over the last decade and that there was no south to north gradient for the frequency of diagnosed cases [32,33]. 

It is noteworthy that we reported a TOSV seroprevalence of 21.2% in Corte (center of Corsica). This is in contrast with the absence of TOSV-seropositive dogs previously reported in the same area in 2013 and 2014 [12]. The lack of seropositive dogs was explained by the sporadic presence of sandflies in the mountainous regions with respect to coasts and by the use of insecticide collars for leishmaniasis prevention [12]. The mobility of the Corsican human population during the summer, especially to the coast, could explain the discrepancy between high rates in humans versus the absence of seropositivity in dogs. 

The major limitation of the present study is the relatively small sample size divided by the district, which could lead to stochastic fluctuations of seroprevalence rates. The reported TOSV seroprevalence of 0% in the district of Sartene (southern) is probably a consequence of the low number of participants included in this district (*n* = 5). The main strength of this study is the serological test used and the virus MN-based assay, the most discriminative serological test adapted to differentiate the affinity of antibodies against different closely related viruses, which reduces the risk of overestimation [34].

To our knowledge, this is the first study that has investigated TOSV and SFSV seroprevalence considering socio-demographic and lifestyle in Corsica. The increasing circulation of TOSV compared to about 10 years ago and its non-homogenous circulation among districts create a potentially significant public health concern for the region, and should encourage the implementation of surveillance systems to control phlebovirus infection. Therefore, entomological studies should be conducted to better understand why there are such differences between the rates observed in the different regions of Corsica. It would be very relevant to consolidate our data with the realization of such a study.

## Figures and Tables

**Table 1:** Description of the population and univariate analysis of factors associated with Toscana virus (TOSV) seropositivity, odds ratio (OR), and *p* value.

Items		All, N	TOSV_Negative, N (%)	TOSV_Positive, N (%)	OR (95% Confidence Intervals (CIs))	*p* (<0.05)
**Total participants**		240	186 (77.5%)	54 (22.5%)		
**Median age (min; max)**		24.5 (9; 84)	24.5 (9; 84)	24 (17; 78)		
**Age groups (years)**	[0,20)	56	45 (80.4)	11 (19.6)	Ref	0.499
	[20,30)	90	73 (81.1)	17 (18.9)	0.95 (0.41; 2.27)	
	[30,40)	30	23 (76.7)	7 (23.3)	1.25 (0.41; 3.6)	
	[40,60)	48	35 (72.9)	13 (27.1)	1.52 (0.61; 3.86)	
	[60,99)	16	10 (62.5)	6 (37.5)	2.45 (0.71; 8.2)	
**Sex**	Female	142	110 (77.5)	32 (22.5)	Ref	0.987
	Male	98	76 (77.5)	22 (22.5)	1 (0.53; 1.84)	
**Residential district**	Calvi	13	6 (46.2)	7 (53.8)	4.33 (1.34; 14.47)	0.005
	Bastia	61	44 (72.1)	17 (27.9)	1.44 (0.71; 2.87)	
	Corte	132	104 (78.8)	28 (21.2)	Ref	
	Ajaccio	29	27 (93.1)	2 (6.9)	0.28 (0.04; 1)	
	Sartène	5	5 (100.0)	0 (0.0)	NA	
**Chronic diseases**	No	201	160 (79.6)	41 (20.4)	1.95 (0.9; 4.08)	0.088
	Yes	39	26 (66.7)	13 (33.3)		
**Organ transplantation**	No	239	185 (77.4)	54 (22.6)	NA	0.475
	Yes	1	1 (100.0)	0 (0.0)		
**Blood transfusion**	No	234	183 (78.2)	51 (21.8)	3.59 (0.65; 19.9)	0.135
	Yes	6	3 (50.0)	3 (50.0)		
**Occupation**	No	68	54 (79.4)	14 (20.6)	1.17 (0.6; 2.38)	0.654
	Yes	172	132 (76.7)	40 (23.3)		
**Travel outside Europe**	No	141	113 (80.1)	28 (19.9)	1.44 (0.78; 2.65)	0.244
	Yes	99	73 (73.7)	26 (26.3)		
**Living in an individual house**	No	111	87 (78.4)	24 (21.6)	1.1 (0.6; 2.03)	0.762
	Yes	129	99 (76.7)	30 (23.3)		
**Living in an apartment**	No	132	103 (78.0)	29 (22.0)	1.07 (0.58; 1.96)	0.828
	Yes	108	83 (76.9)	25 (23.1)		
**Highest degree or level of school**						
***No school qualification***	No	230	181 (78.7)	49 (21.3)	3.69 (0.99;13.78)	0.051
	Yes	10	5 (50.0)	5 (50.0)		
***General certificate of secondary education***	No	219	173 (79.0)	46 (21.0)	2.31 (0.87; 5.83)	0.090
	Yes	21	13 (61.9)	8 (38.1)		
***High school diploma***	No	140	103 (73.6)	37 (26.4)	0.57 (0.29; 1.07)	0.081
	Yes	100	83 (83.0)	17 (17.0)		
***Bachelor's degree 2***	No	218	167 (76.6)	51 (23.4)	0.52 (0.12; 1.59)	0.271
	Yes	22	19 (86.4)	3 (13.6)		
***Bachelor's degree 3 or more***	No	154	119 (77.3)	35 (22.7)	0.96 (0.5; 1.8)	0.910
	Yes	86	67 (77.9)	19 (22.1)		
**Hunting**	No	214	168 (78.5)	46 (21.5)	1.62 (0.63; 3.86)	0.301
	Yes	26	18 (69.2)	8 (30.8)		
**Breeding**	No	228	176 (77.2)	52 (22.8)	0.68 (0.1;2.67)	0.608
	Yes	12	10 (83.3)	2 (16.7)		
**Farmer**	No	236	182 (77.1)	54 (22.9)	NA	0.151
	Yes	4	4 (100.0)	0 (0.0)		

**Table 2:** Multivariate analysis of factors associated with Toscana virus seropositivity.

Items	OR (95% CIs)	*p* Value
**District of Calvi**	4.33 (1.34; 14.47)	0.005
**District of Bastia**	1.44 (0.71; 2.87)	
**District of Ajaccio**	0.28 (0.04; 1)	
**District of Sartene**	0	

## References

[1] Depaquit J., Grandadam M., Fouque F., Andry P.E., Peyrefitte C. (2010). Arthropod-borne viruses transmitted by Phlebotomine sandflies in Europe: A review. Eurosurveillance.

[2] Alkan C., Bichaud L., de Lamballerie X., Alten B., Gould E.A., Charrel R.N. (2013). Sandfly-borne phleboviruses of Eurasia and Africa: Epidemiology, genetic diversity, geographic range, control measures. Antivir. Res..

[3] Charrel R.N., Bichaud L., de Lamballerie X. (2012). Emergence of Toscana virus in the mediterranean area. World J. Virol..

[4] Dionisio D., Esperti F., Vivarelli A., Valassina M. (2003). Epidemiological, clinical and laboratory aspects of sandfly fever. Curr. Opin. Infect. Dis..

[5] Dobler G., Treib J., Haass A., Frosner G., Woesner R., Schimrigk K. (1997). Toscana virus infection in German travellers returning from the Mediterranean. Infection.

[6] Nougairede A., Bichaud L., Thiberville S.D., Ninove L., Zandotti C., de Lamballerie X., Brouqui P., Charrel R.N. (2013). Isolation of Toscana virus from the cerebrospinal fluid of a man with meningitis in Marseille, France, 2010. Vector Borne Zoonotic Dis..

[7] Marlinge M., Crespy L., Zandotti C., Piorkowski G., Kaphan E., Charrel R.N., Ninove L. (2014). Afebrile meningoencephalitis with transient central facial paralysis due to Toscana virus infection, southeastern France, 2014 [corrected]. Eurosurveillance.

[8] Mosnier E., Charrel R., Vidal B., Ninove L., Schleinitz N., Harle J.R., Bernit E. (2013). Toscana virus myositis and fasciitis. Med. Mal. Infect..

[9] Bichaud L., Piarroux R.P., Izri A., Ninove L., Mary C., De Lamballerie X., Charrel R.N. (2011). Low seroprevalence of sandfly fever Sicilian virus antibodies in humans, Marseille, France. Clin. Microbiol. Infect..

[10] Bichaud L., Izri A., de Lamballerie X., Moureau G., Charrel R.N. (2014). First detection of Toscana virus in Corsica, France. Clin. Microbiol. Infect..

[11] De Lamballerie X., Tolou H., Durand J.P., Charrel R.N. (2007). Prevalence of Toscana virus antibodies in volunteer blood donors and patients with central nervous system infections in southeastern France. Vector Borne Zoonotic Dis..

[12] Dahmani M., Alwassouf S., Grech-Angelini S., Marie J.L., Davoust B., Charrel R.N. (2016). Seroprevalence of Toscana virus in dogs from Corsica, France. Parasit. Vectors.

[13] Flahault A., Blanchon T., Dorleans Y., Toubiana L., Vibert J.F., Valleron A.J. (2006). Virtual surveillance of communicable diseases: A 20-year experience in France. Stat. Methods Med. Res..

[14] Alwassouf S., Christodoulou V., Bichaud L., Ntais P., Mazeris A., Antoniou M., Charrel R.N. (2016). Seroprevalence of Sandfly-Borne Phleboviruses Belonging to Three Serocomplexes (Sandfly fever Naples, Sandfly fever Sicilian and Salehabad) in Dogs from Greece and Cyprus Using Neutralization Test. PLoS Negl. Trop. Dis..

[15] Pierro A., Ficarelli S., Ayhan N., Morini S., Raumer L., Bartoletti M., Mastroianni A., Prati F., Schivazappa S., Cenni P. (2017). Characterization of antibody response in neuroinvasive infection caused by Toscana virus. Clin. Microbiol. Infect..

[16] Ayhan N., Sherifi K., Taraku A., Berxholi K., Charrel R.N. (2017). High Rates of Neutralizing Antibodies to Toscana and Sandfly Fever Sicilian Viruses in Livestock, Kosovo. Emerg. Infect. Dis..

[17] Pourhoseingholi M.A., Vahedi M., Rahimzadeh M. (2013). Sample size calculation in medical studies. Gastroenterol. Hepatol. Bed Bench.

[18] Calamusa G., Valenti R.M., Vitale F., Mammina C., Romano N., Goedert J.J., Gori-Savellini G., Cusi M.G., Amodio E. (2012). Seroprevalence of and risk factors for Toscana and Sicilian virus infection in a sample population of Sicily (Italy). J. Infect..

[19] R Development Core Team (2005). R: A Language and Environment for Statistical Computing.

[20] Charrel R.N., Gallian P., Navarro-Mari J.M., Nicoletti L., Papa A., Sanchez-Seco M.P., Tenorio A., de Lamballerie X. (2005). Emergence of Toscana virus in Europe. Emerg. Infect. Dis..

[21] Echevarria J.M., de Ory F., Guisasola M.E., Sanchez-Seco M.P., Tenorio A., Lozano A., Cordoba J., Gobernado M. (2003). Acute meningitis due to Toscana virus infection among patients from both the Spanish Mediterranean region and the region of Madrid. J. Clin. Virol..

[22] Papa A., Konstantinou G., Pavlidou V., Antoniadis A. (2006). Sandfly fever virus outbreak in Cyprus. Clin. Microbiol. Infect..

[23] Marchi S., Trombetta C.M., Kistner O., Montomoli E. (2017). Seroprevalence study of Toscana virus and viruses belonging to the Sandfly fever Naples antigenic complex in central and southern Italy. J. Infect. Public Health.

[24] Francisci D., Papili R., Camanni G., Morosi S., Ferracchiato N., Valente M., Ciufolini M.G., Baldelli F. (2003). Evidence of Toscana virus circulation in Umbria: First report. Eur. J. Epidemiol..

[25] Terrosi C., Olivieri R., Bianco C., Cellesi C., Cusi M.G. (2009). Age-dependent seroprevalence of Toscana virus in central Italy and correlation with the clinical profile. Clin. Vaccine Immunol..

[26] Jaijakul S., Arias C.A., Hossain M., Arduino R.C., Wootton S.H., Hasbun R. (2012). Toscana meningoencephalitis: A comparison to other viral central nervous system infections. J. Clin. Virol..

[27] Navarro J.M., Fernandez-Roldan C., Perez-Ruiz M., Sanbonmatsu S., de la Rosa M., Sanchez-Seco M.P. (2004). Meningitis by Toscana virus in Spain: Description of 17 cases. Med. Clin. (Barc.).

[28] Charrel R., Gallian P., de Lamballerie X. (2011). Unité des Virus Emergents (UVE: Aix Marseille Univ, IRD 190, INSERM 1207, IHU Méditerranée Infection).

[29] Rioux J.A., Houin R., Leger N., Croset H., Deniau M., Poinsot S. (1971). New localizations in Corsica of Phlebotomus sergenti Parrot, 1917. Ann. Parasitol. Hum. Comp..

[30] Naucke T.J., Menn B., Massberg D., Lorentz S. (2008). Winter activity of Phlebotomus (Transphlebotomus) mascittii, Grassi 1908 (Diptera: Psychodidae) on the island of Corsica. Parasitol. Res..

[31] Chamaille L., Tran A., Meunier A., Bourdoiseau G., Ready P., Dedet J.P. (2010). Environmental risk mapping of canine leishmaniasis in France. Parasit. Vectors.

[32] Bourdeau P. (2007). La Leishmaniose gagne du terrain en France.

[33] Martinetti L. (2013). Dépistage, Traitement et Prévention de la Leishmaniose Canine en Corse: Enquête Auprès des Vétérinaires Praticiens de L’île. Thèse d’exercice Médecine vétérinaire.

[34] Cusi M.G., Savellini G.G. (2011). Diagnostic tools for Toscana virus infection. Expert Rev. Anti Infect. Ther..

